# Effects of a tart cherry (*Prunus cerasus* L.) phenolic extract on *Porphyromonas gingivalis* and its ability to impair the oral epithelial barrier

**DOI:** 10.1371/journal.pone.0246194

**Published:** 2021-01-26

**Authors:** Amel Ben Lagha, Geneviève Pellerin, Katy Vaillancourt, Daniel Grenier

**Affiliations:** Oral Ecology Research Group, Faculty of Dentistry, Université Laval, Quebec City, QC, Canada; Hungarian Academy of Sciences, HUNGARY

## Abstract

Periodontal diseases, including gingivitis and periodontitis, are a global oral health problem. *Porphyromonas gingivalis*, a key pathogen involved in the onset of periodontitis, is able to colonize the subgingival epithelium and invade the underlying connective tissue due to the contribution of cysteine proteases known as gingipains. In this study, we investigated the effects of a phenolic extract prepared from tart cherry (*Prunus cerasus* L.) juice on the growth, adherence, and protease activity of *P*. *gingivalis*. We also assessed the protective effect of the tart cherry extract on the disruption of the oral epithelial barrier induced by *P*. *gingivalis*. The tart cherry extract that contains procyanidins and quercetin and its derivatives (rutinoside, glucoside) as the most important phenolic compounds attenuated *P*. *gingivalis* growth, reduced adherence to an experimental basement membrane matrix model, and decreased the protease activities of *P*. *gingivalis*. The tart cherry extract also exerted a protective effect on the integrity of the oral epithelial barrier in an *in vitro* model infected with *P*. *gingivalis*. More specifically, the extract prevented a decrease in transepithelial electrical resistance as well as the destruction of tight junction proteins (zonula occludens-1 and occludin). These results suggest that the tart cherry phenolic extract may be a promising natural product for the treatment of periodontitis through its ability to attenuate the virulence properties of *P*. *gingivalis* and curtail the ability of this pathogen to impair the oral epithelial barrier.

## Introduction

Over 700 microbial species have been detected in the oral cavity, the majority of which maintain commensal relationships with the host [1]. However, when oral hygiene is deficient, dental biofilm accumulates and develops into a complex microbial community with synergistic interactions leading to metabolic collaborations, nutritional interdependence, and oxygen consumption [1]. This enables the establishment of obligate anaerobic bacteria, some of which have the ability to modulate the host inflammatory response [2]. The disruption of oral homeostasis induces a dysbiosis in the microbial community that triggers the onset of periodontitis, an inflammatory disease of bacterial origin characterized by the destruction of the underlying structures of the periodontium [3]. If left untreated, this can lead to extensive destruction of connective tissue, resorption of alveolar bone, and tooth loss. It is estimated that nearly 10% of the adult population worldwide is afflicted by severe periodontitis [4]. Over the last decade, epidemiologic evidence has accumulated suggesting that periodontal disease is a risk factor for more serious systemic diseases, including cardiovascular disease, type 2 diabetes, and rheumatoid arthritis [5].

The most documented periodontal pathogen is likely *Porphyromonas gingivalis*, a Gram-negative anaerobic bacterium that produces a broad array of virulence factors, including proteases [6]. *P*. *gingivalis* expresses three different cysteine proteases (Arg-gingipains A and B and Lys-gingipain) in cell membrane-bound and secreted forms [7, 8]. These proteases are involved in nutrient acquisition, host colonization, inactivation of host defense mechanisms, and tissue destruction [7, 8]. Therefore, gingipains are likely to be critical for bacterial survival and multiplication *in vivo*. In addition, *P*. *gingivalis* lipopolysaccharides (LPS) stimulate cytokine and matrix metalloproteinase production by resident and immune cells in the periodontium [9]. These inflammatory mediators play a prominent role in the pathogenesis of periodontitis by mediating periodontal attachment loss and alveolar bone destruction [10–13].

There has been a growing interest in tart cherry (*Prunus cerasus* L.) research over the last decade due to accumulating evidence that it is a functional food. Studies have suggested that whole fruits as well as extracts can lower the risk of cardiovascular disease, decrease low-density lipoprotein (LDL) cholesterol levels, help manage type 2 diabetes, and reduce inflammatory disorders such as arthritis [14–17]. Consuming tart cherry juice also reduces recovery time and soreness after resistance or endurance training by athletes by lowering oxidative stress and muscle inflammation [18]. These health benefits have been strongly linked to its high polyphenol (mainly flavonoid) content [19]. The potential of polyphenols from berry fruits for the prevention and treatment of periodontal diseases has been widely explored more recently [20–22]. Proanthocyanidins from blueberry and cranberry have been shown to inhibit biofilm formation and the adherence of major periodontal pathogens, exert anti-inflammatory properties, and reinforce epithelial barrier integrity [20–22]. Recent work in our laboratory showed that two tart cherry phenolic extracts exhibit anti-adherence properties and impede biofilm formation by the major oral pathogens *Streptococcus mutans*, *Candida albicans*, and *Fusobacterium nucleatum* [23]. The aim of the present study was to investigate the effects of a phenolic extract prepared from tart cherry (*P*. *cesarus* L.) juice on the growth, adherence, and protease activity of *P*. *gingivalis*. We also assessed the ability of the tart cherry extract to protect against the *P*. *gingivalis*-induced disruption of the oral epithelial barrier.

## Materials and methods

### Preparation of the tart cherry phenolic extract

Montmorency tart cherry (*P*. *cerasus* L.) juice concentrate kindly provided by King Orchards (Central Lake, MI, USA) underwent extensive dialysis (3 days/4°C) through a membrane with a 1-kDa molecular weight cut-off. Non-dialyzable material was freeze-dried and stored at 4°C in the dark. The phenolic composition of the tart cherry extract determined by chromatographic and mass spectrometry analyses has been reported previously [23]. Quercetin and its derivatives (rutinoside, glucoside) as well as procyanidins are the main constituents of the extract.

### Bacteria and growth conditions

*P*. *gingivalis* ATCC 33277 was grown under anaerobic conditions (80% N_2_, 10% CO_2_, 10% H_2_) at 37°C in Todd-Hewitt Broth (THB; BBL Microbiology Systems, Cockeysville, MD, USA) supplemented with 0.001% hemin (Sigma-Aldrich Canada Co., Oakville, ON, Canada) and 0.0001% vitamin K (Sigma-Aldrich Canada Co.) (THB-HK).

### Effect of the tart cherry extract on *P*. *gingivalis* growth

A 24-h culture (early stationary growth phase) of *P*. *gingivalis* was diluted in fresh culture medium to an optical density at 660 nm (OD_660_) of 0.1. Equal volumes (100 μL) of diluted bacterial culture and two-fold serial dilutions of the tart cherry extract (ranging from 0.49 to 2000 μg/mL) in THB-HK were added to the wells of a 96-well tissue culture microplate (Sarstedt Inc., St-Leonard, QC, Canada). The microplate was incubated at 37°C for 48 h under anaerobic conditions prior to assessing bacterial growth by recording the OD_660_ using a Synergy 2 microplate reader (BioTek Instruments, Winooski, VT, USA). Wells with no tart cherry extract or *P*. *gingivalis* were used as controls. Triplicate assays in two independent experiments were performed.

### Iron-chelating activity of the tart cherry extract

The capacity of the tart cherry extract to chelate iron was determined using a universal siderophore assay, with chrome azurol S and hexadecyltrimethylammonium bromide as indicators, as per the protocol described by Schwyn and Neilands [24]. Ferrichrome (Sigma-Aldrich Canada Co.), a siderophore produced by *Ustilago sphaerogena*, served as a positive control. Triplicate assays in two independent experiments were performed.

### Effect of the tart cherry extract on the adherence of *P*. *gingivalis* to a basement membrane matrix model

To determine the effect of the tart cherry extract on the adherence of *P*. *gingivalis* to an experimental basement membrane matrix model, the bacterial cells were first labeled with fluorescein isothiocyanate (FITC) as described previously [25]. Matrigel™ (BD Biosciences, San Jose, CA, USA), a solubilized basement membrane preparation extracted from the Engelbreth-Holm-Swarm mouse sarcoma composed of several extracellular matrix proteins, including laminin, type IV collagen, heparin sulfate proteoglycans, and entactin, was diluted 1/10 in ice-cold PBS and was added (50 μL) to the wells of a 96-well clear bottom black microplate (Greiner Bio-One North America, Monroe, NC, USA). After gelification (room temperature for 2 h), the Matrigel™ was washed twice with PBS, and two-fold serial dilutions of the tart cherry extract (100 μL; 31.25, 62.5, 125, or 250 μg/mL in PBS) were added on top of the gel. After 30 min, 100 μL of FITC-labeled *P*. *gingivalis* cells (OD_660_ = 0.5) were added to the wells, and the plate was incubated for a further 4 h at 37°C. Unbound bacteria were then removed by aspiration, and the wells were washed twice with PBS. Relative fluorescence units (RFU; excitation wavelength 495 nm; emission wavelength 525 nm) corresponding to the level of bacterial adherence were determined using a Synergy 2 microplate reader. Wells with no bacteria were used as controls to measure basal auto-fluorescence. Control wells without tart cherry extract were used to determine 100% adherence values. Triplicate assays in two independent experiments were performed.

### Effect of the tart cherry extract on the protease activities of *P*. *gingivalis*

The effect of the tart cherry extract on collagen degradation by *P*. *gingivalis* was first investigated. A 48-h cell-free culture supernatant was mixed with the tart cherry extract (final concentration of 31.25, 62.5, 125, or 250 μg/mL) and fluorescent substrate type I collagen DQ (Molecular Probes, Eugene, OR, USA) (100 μg/mL). Leupeptin (1 μM; Sigma-Aldrich Canada Co.) served as a positive inhibitor control. The mixtures were incubated at 37°C, and fluorescence was measured every 30 min for 2 h using a Synergy 2 multi-mode microplate reader with the excitation and emission wavelengths set at 495 nm and 525 nm, respectively. The effect of the tart cherry extract on *P*. *gingivalis* Arg- and Lys-gingipains was also assessed. Briefly, *P*. *gingivalis* cells suspended in 50 mM phosphate-buffered saline (PBS, pH 7.2) at an OD_660_ of 0.1 were mixed with the tart cherry extract (final concentration of 31.25, 62.5, 125, or 250 μg/mL), 10 mM dithiothreitol, and either 5 mM N-α-benzoyl-DL-arginine-*p*-nitroanilide (Arg-gingipain substrate; Sigma-Aldrich Canada Co.) or N-*p*-tosyl-glycine-proline-lysine-*p*-nitroanilide (Lys-gingipain substrate; Sigma-Aldrich Canada Co.). Nα-*p*-tosyl-L-lysine chloromethyl ketone hydrochloride (8 mM; Sigma-Aldrich Canada Co.) served as a positive inhibitor control. After a 60-min incubation at 37°C, the hydrolysis of the chromogenic substrates was determined by recording the absorbance at 405 nm (A_405_) with a Synergy 2 multi-mode microplate reader. Triplicate assays in two independent experiments were performed.

### Protective effect of the tart cherry extract against the *P*. *gingivalis*-induced disruption of oral epithelial barrier integrity in an *in vitro* model

The immortalized oral epithelial cell line B11, previously characterized by Groeger, Michel & Meyle [26], was cultured at 37°C in a 5% CO_2_ atmosphere in keratinocyte-serum free medium (K-SFM) supplemented with growth factors (50 μg/mL of bovine pituitary extract and 5 ng/mL of human epidermal growth factor) and 100 μg/mL of penicillin G-streptomycin. The ability of the tart cherry extract to preserve the integrity of the oral epithelial barrier of an *in vitro* model infected with *P*. *gingivalis* was investigated by monitoring the transepithelial electrical resistance (TEER). B11 cells (3 × 10^5^ cells per insert) were seeded on Costar Transwell clear polyester membrane inserts (6.5-mm diameter; 0.4-μm pore size; Corning Co., Cambridge, MA, USA). The apical and basolateral compartments were filled with 100 μL and 600 μL of complete K-SFM, respectively. Following a 72-h incubation (37°C, 5% CO_2_ atmosphere), the conditioned medium was replaced with fresh antibiotic-free K-SFM, and the cells were incubated for a further 16 h. Non-cytotoxic concentrations of the tart cherry extract (7.813, 15.625, 31.25, 62.5, or 125 μg/mL in culture medium), determined in a preliminary analysis using a 3-(4,5-dimethylthiazol-2-yl)-2,5diphenyltetrazolium bromide (MTT) assay and a 48-h exposure (Roche Diagnostics, Laval, QC, Canada), were added to the apical compartment along with *P*. *gingivalis* at a multiplicity of infection (MOI) of 10^4^. TEER values were measured with an ohm/voltmeter (EVOM2; World Precision Instruments, Sarasota, FL, USA) after a 1, 2, 4, 6, 8, 24, and 48-h incubation. Measurements were recorded in triplicate for each treatment. The results were converted to Ohms (Ω)/cm^2^ by multiplying the resistance by the membrane surface area. Each value was compared to the initial resistance of the well at time 0 (100% value). The integrity of the oral epithelial barrier in the presence of *P*. *gingivalis* and the tart cherry extract was also investigated by tracking the paracellular transport of FITC-conjugated dextran (FD-4; 4.4 kDa; Sigma-Aldrich Canada Co.). Briefly, B11 cells were seeded as described above, and FD-4 (1 mg/mL), *P*. *gingivalis* (MOI of 10^4^), and the tart cherry extract (15.625, 31.25, 62.5, or 125 μg/mL) were added to the apical compartment. Fluorescence in the basolateral compartment was measured using a Synergy 2 multi-mode microplate reader after a 6, 24, and 48-h incubation. All conditions were tested in triplicate.

### Immunofluorescence staining of zonula occludens-1 and occludin

The effect of the tart cherry extract on *P*. *gingivalis*-mediated damage to the tight junction proteins zonula occludens-1 (ZO-1) and occludin was monitored by immunofluorescence staining. B11 cells were treated (24 h) with *P*. *gingivalis* (MOI of 10^4^). The tight junction proteins were immunostained with either occludin antibody-Alexa Fluor 488 conjugate or ZO-1 antibody-Alexa Fluor 594 conjugate (Thermo Fisher Scientific, Waltham, MA, USA), as described previously [23]. An Olympus FSX100 fluorescence microscope and FSX-BSW imaging software (Olympus, Tokyo, Japan) were used to observe the immunostaining. Treatments were performed in triplicate and a representative set of data is presented.

### Statistical analysis

Results are expressed as means ± standard deviations (SD). The statistical analyses were performed using a one-way analysis of variance with a post hoc Bonferroni multiple comparison test (GraphPad Software Inc., La Jolla, CA, USA). The level of significance was set at *p* < 0.01.

## Results

**Fig 1:**
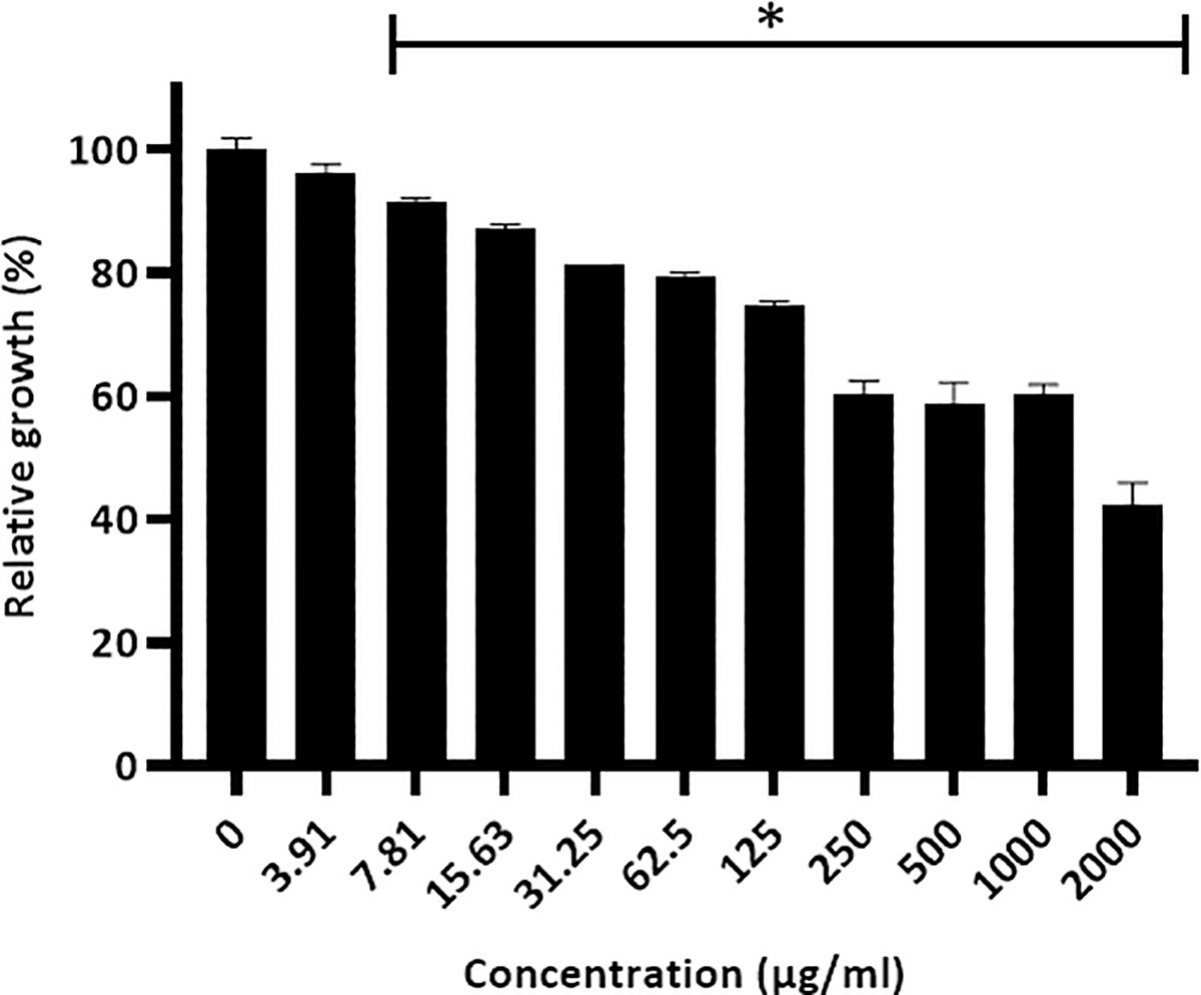
Effect of the tart cherry extract on the growth of *P*. *gingivalis*. A 100% value was attributed to the control (no tart cherry extract). Results are expressed as the means ± SD of triplicate assays from two independent experiments. *, Significantly different (*p* < 0.01) from the control (no tart cherry extract).

**Fig 2:**
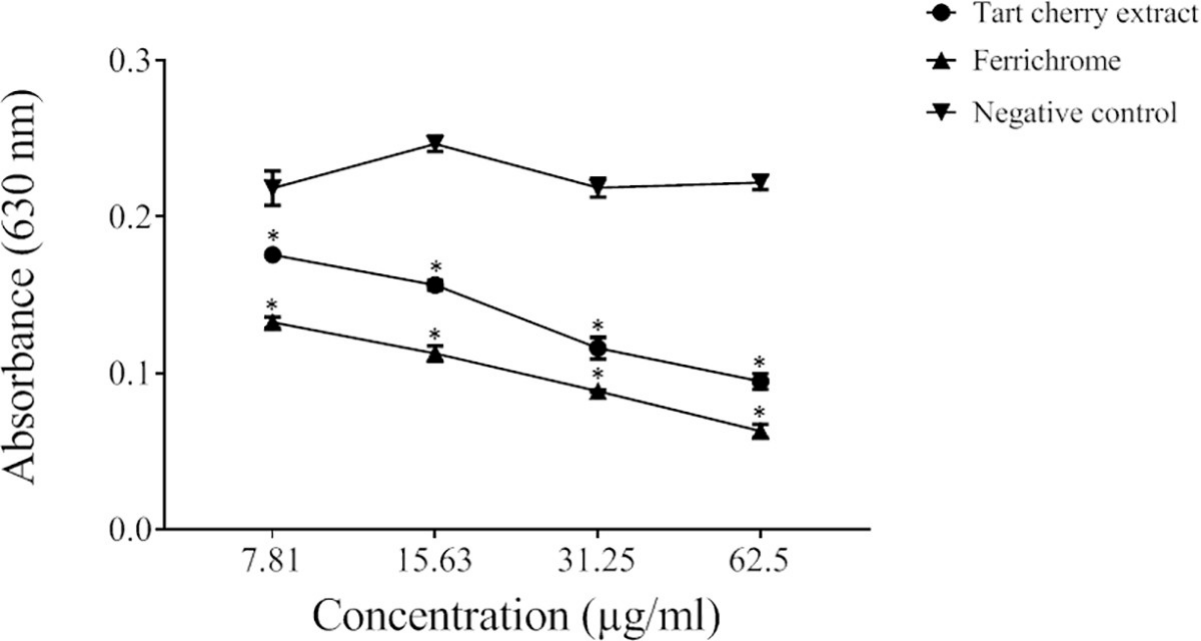
Iron-chelating activity of the tart cherry extract assessed using a universal siderophore colorimetric assay. Results are expressed as the means ± SD of triplicate assays from two independent experiments. A decrease in A_630_ occurs when a strong chelator removes the iron from the chrome azurol S dye. Ferrichrome, a siderophore produced by *U*. *sphaerogena*, was used as positive control. All values are significantly different (*p* < 0.01) from the negative control (no tart cherry extract).

The antibacterial activity of the tart cherry extract against *P*. *gingivalis* was determined by assessing bacterial growth in a broth microdilution assay following a 48-h incubation. As shown in Fig 1, although the tart cherry extract did not completely inhibit growth, a significant reduction in growth was observed starting with a concentration of 7.81 μg/mL of the tart cherry extract. At the highest concentration tested (2 mg/mL), the tart cherry extract reduced the growth of *P*. *gingivalis* by 57.3%. We then used a universal siderophore assay to assess the iron-chelating activity of the tart cherry extract, a property that may contribute to its ability to reduce the growth of *P*. *gingivalis*. Fig 2 shows that the tart cherry extract dose-dependently chelated iron as did ferrichrome, which was used as a positive control.

The effect of the tart cherry extract on the adherence of FITC-labeled *P*. *gingivalis* to a polystyrene surface coated with Matrigel^®^, used as an experimental basement membrane matrix model, was investigated. The tart cherry extract dose-dependently inhibited the adherence of *P*. *gingivalis* to the Matrigel^®^. More specifically, at a concentration of 250 μg/mL, the extract reduced adherence by 35.0% (Table 1).

**Table 1:** Effect of the tart cherry extract on the adherence of *P*. *gingivalis* to Matrigel^®^, a basement membrane matrix model.

Tart cherry extract (μg/mL)	Relative adherence (%)
0	100 ± 7.8
31.25	94.6 ± 4.5
62.5	99.3 ± 15.6
125	67.7 ± 14.3 *
250	65.0 ± 10.1 *

Results are expressed as the means ± SD of triplicate assays from two independent experiments.

*, Significantly different (*p* < 0.01) from the control (no tart cherry extract).

**Fig 3:**
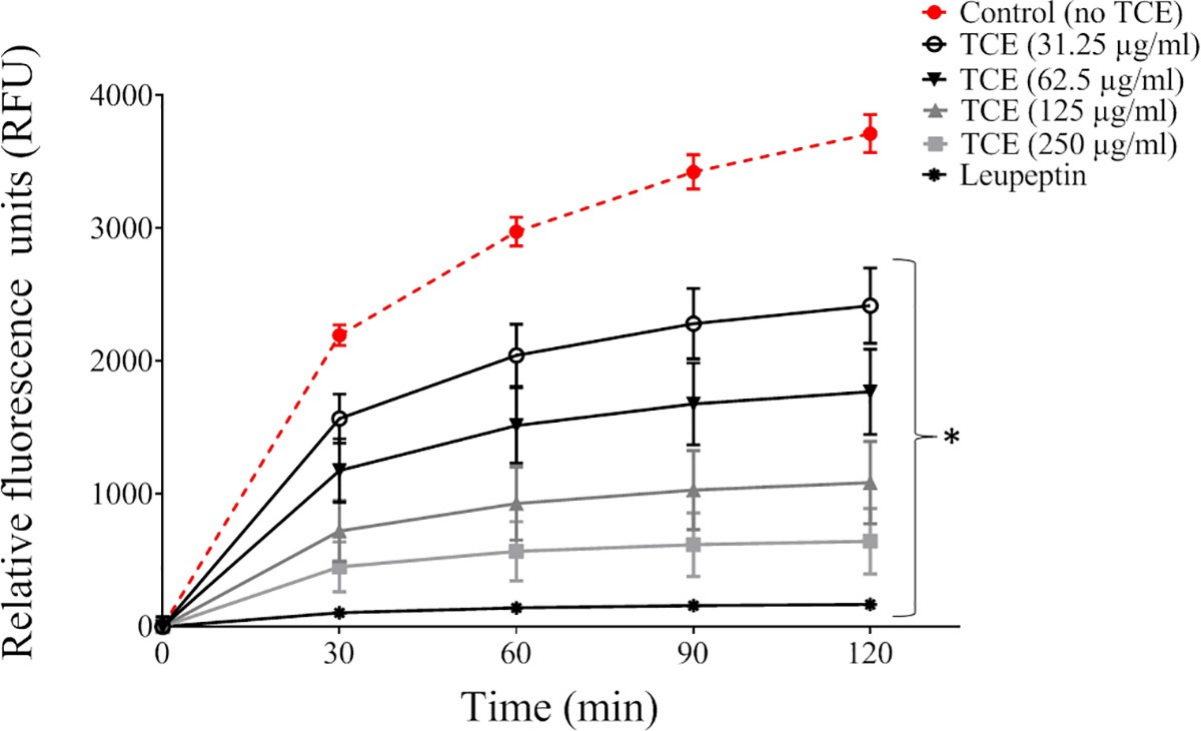
Effect of the Tart Cherry Extract (TCE) on collagen degradation by *P*. *gingivalis*. Collagen degradation was assayed using a fluorescent substrate. Results are expressed as the means ± SD of triplicate assays.

*P*. *gingivalis* can mediate the destruction of periodontal connective tissue by breaking down type I collagen. We showed that the tart cherry extract inhibits, in a dose-and time-dependent manner, the degradation of collagen by *P*. *gingivalis*. After a 2-h incubation, the lowest concentration (31.25 μg/mL) of the extract inhibited collagen degradation by 33.3% while the highest concentration (250 μg/mL) inhibited degradation by 82.1% (Fig 3). The effect of the tart cherry extract on the cell-associated Arg- and Lys-gingipain activities of *P*. *gingivalis* was also evaluated. As reported in Table 2, the extract dose-dependently inhibited both activities. More specifically, at a concentration of 250 μg/mL, the extract inhibited the Arg- and Lys-gingipains by 62.5% and 37.5%, respectively.

**Table 2:** Effect of the tart cherry extract on the Arg- and Lys-gingipain activities of *P*. *gingivalis*.

Compounds	Arg-gingipain activity (%)	Lys-gingipain activity (%)
None	100	100
Tart cherry extract (μg/mL)		
31.25	100 ± 12.54	97.94 ± 5.51
62.5	86.73 ± 4.55 *	92.03 ± 2.51
125	58.41 ± 4.54 *	83.29 ± 5.55 *
250	37.47 ± 11.81 *	62.47 ± 7.40 *
TLCK (commercial inhibitor)	0 *	0 *

Results are expressed as the means ± SD of triplicate assays from two independent experiments.

*, Significantly different (*p* < 0.01) from the control (no tart cherry extract).

**Fig 4:**
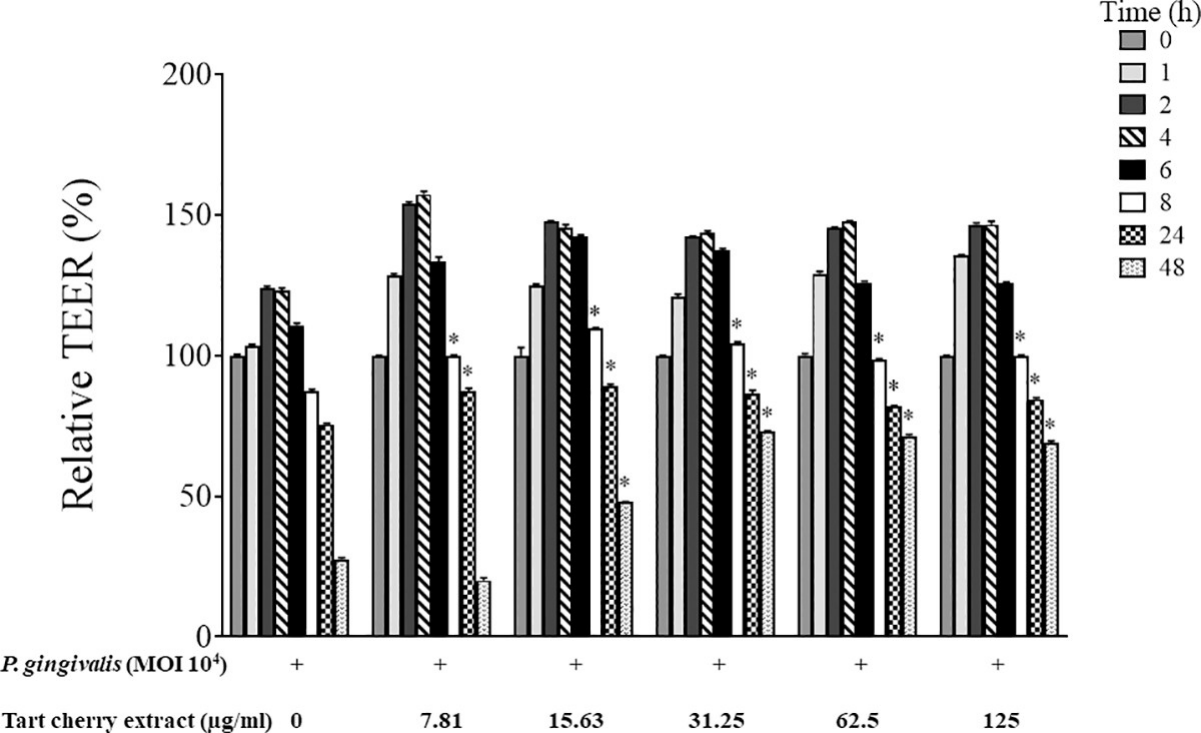
Effect of the tart cherry extract on the disruption of epithelial barrier integrity by *P*. *gingivalis* (MOI of 10^4^) assessed by monitoring TEER over a period of 48 h. A 100% value was attributed to the TEER value at time 0. Results are expressed as the means ± SD of triplicate assays. *, Significantly different (*p* < 0.01) from the control (no tart cherry extract).

As shown in Fig 4, for an incubation time ≥ 8 h, *P*. *gingivalis* (MOI of 10^4^) caused a significant time-dependent decrease in TEER in an *in vitro* model of the oral epithelial barrier using a double-chamber system. The increase in TEER observed for shorter incubation periods (1, 2, 4, and 6 h) may be an epithelial cell barrier defense response to the initial bacterial challenge. The TEER values decreased by 21.8%, 32.5%, and 75.6% after a 8, 24, and 48-h exposure to *P*. *gingivalis*, respectively. However, the addition of the tart cherry extract to the apical compartment together with *P*. *gingivalis* resulted in a significant protective effect with as little as 7.9 μg/mL following a 8 and 24-h incubation, as indicated by the smaller decrease in TEER values. Concentrations of the tart cherry extract ≥ 15.625 μg/mL were required to prevent the *P*. *gingivalis*-mediated decrease in TEER following a 48-h incubation.

**Fig 5:**
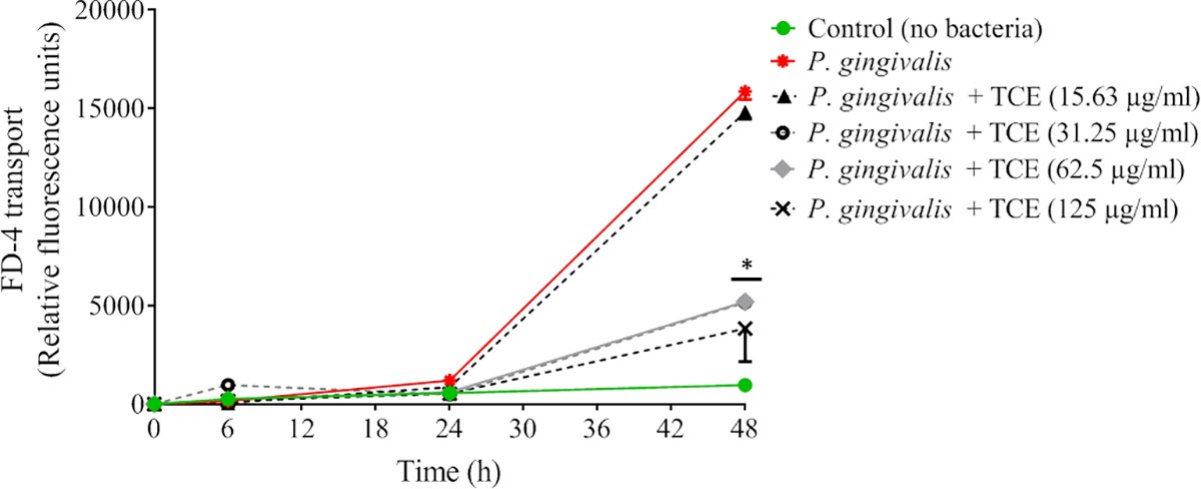
Effect of the tart cherry extract on the disruption of epithelial barrier integrity by *P*. *gingivalis* (MOI of 10^4^) assessed by measuring FD-4 transepithelial transport over a period of 48 h. Results are expressed as the means ± SD of triplicate assays. *, Significantly different (*p* < 0.01) from the control (no tart cherry extract).

*P*. *gingivalis*-mediated damage to the oral epithelial barrier and the protective effect of the tart cherry extract were further investigated by monitoring FD-4 transport across the *in vitro* model. No significant differences for all the concentrations of the extract tested were observed after a 24-h challenge with *P*. *gingivalis* (Fig 5). However, after a 48-h challenge, the tart cherry extract decreased the *P*. *gingivalis*-mediated flux of FD-4 across the oral epithelial barrier. At a concentration of 125 μg/mL, the tart cherry extract reduced the *P*. *gingivalis*-mediated flux of FD-4 by 4.2-fold.

**Fig 6:**
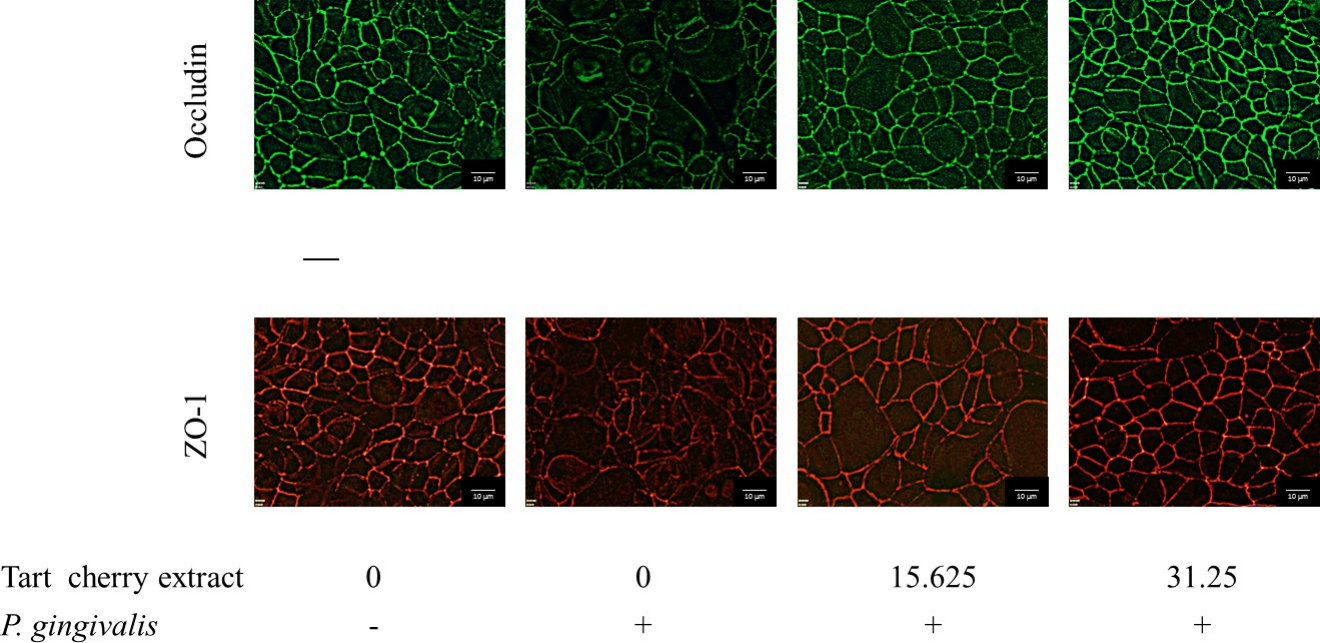
Effect of the tart cherry extract on the immunofluorescence staining of zonula-occludens 1 (ZO-1) and occludin of oral epithelial cell monolayers treated with *P*. *gingivalis* (MOI of 10^4^) for 24 h.

ZO‐1 and occludin in the *P*. *gingivalis*-treated oral epithelial barrier model were immunostained to investigate the effect of the tart cherry extract on these two tight junction proteins. Treating the oral epithelial barrier model with *P*. *gingivalis* resulted in discontinuous, weak labeling of ZO‐1 and occludin compared to untreated control cells (Fig 6). However, the addition of ≥ 31.25 μg/mL of the tart cherry extract together with *P*. *gingivalis* prevented the degradation of ZO‐1 and occludin.

## Discussion

*P*. *gingivalis* is a key member of the pathogenic subgingival biofilm that induces chronic inflammation and that, in turn, leads to destructive periodontitis [6]. It has been detected in 75% of active sites and in 59.7% of inactive sites in 96% of patients with progressive adult periodontitis [27]. Given its critical role in the pathogenesis of periodontitis, *P*. *gingivalis* may be a potential target for developing a therapeutic approach to prevent and treat this disease. In this study, we investigated the potential of a tart cherry phenolic extract, containing as main components procyanidins as well as quercetin and its derivatives (rutinoside, glucoside), to help restore periodontal health by focusing on its effect on *P*. *gingivalis*.

We first showed that the growth of *P*. *gingivalis* is attenuated by the tart cherry extract, which may be associated, at least in part, with the fact that the extract can chelate iron. Iron is an essential nutrient for most bacteria and, as such, is an important factor during infections [28]. Hemin was added to the culture medium as a source of iron and protoporphyrin IX in order to support the *in vitro* growth of *P*. *gingivalis*. Ohya *et al*. have reported that the growth of *P*. *gingivalis* is negatively affected under hemin-limited conditions [29]. Given this, the curtailment of iron available to *P*. *gingivalis* due to the iron-chelating property of the tart cherry extract could explain the significant reduction in growth. Interestingly, other studies have shown that plant polyphenols act as natural antimicrobial compounds because of their ability to chelate iron ions [30, 31] and that the iron-chelating efficiency of phenolic compounds depends on the number of binding sites they possess [32].

The establishment of a dysbiotic periodontal community, of which *P*. *gingivalis* is a key member, is largely dependent on its ability to obtain nutrients (peptides, amino acids, iron) from the proteolytic degradation of host proteins such as tissue constituents (collagen, fibronectin, etc.) and hemoproteins (hemoglobin, hemopexin, etc.) [4]. We thus explored the ability of the tart cherry extract to inhibit the proteases produced by *P*. *gingivalis*. The tart cherry extract acted as an effective inhibitor of Arg-gingipains and, to a lesser extent, of Lys-gingipain. In addition to its iron-scavenging property, the capacity of the tart cherry extract to inhibit Arg- and Lys-gingipain activities may also contribute to attenuating the growth of *P*. *gingivalis*. The capacity of other plant polyphenols, such as those in black tea, green tea, blueberry, and cranberry, to inhibit *P*. *gingivalis* gingipains has been previously reported [25, 33, 34].

A variety of pathogenic roles have been associated with Arg-gingipains A and B and Lys-gingipain produced by *P*. *gingivalis* [7, 8]. Gingipains enable bacteria to evade the innate immunity response in periodontal pockets by reducing macrophage phagocytosis by decreasing the surface expression of CD14 [35]. Moreover, gingipains may play a key role in allowing *P*. *gingivalis* to survive complement-dependent killing [36, 37]. The inhibition of gingipains by the tart cherry extract may thus prevent *P*. *gingivalis*, along with other periodontal pathogens, to escape the host defense system and thus enhance its survival in periodontal pockets.

As *P*. *gingivalis* gingipains are considered to play a central role in the pathogenesis of periodontitis, there is considerable interest in the possibility of identifying and developing gingipain inhibitors as potential therapeutics [38]. Such inhibitors may have applications beyond periodontal diseases. Indeed, *P*. *gingivalis* gingipains were recently detected in the brains of Alzheimer's patients where they may exert a neurotoxic effect associated with neuronal dysfunction [39]. In addition, it has been suggested that the blockage of this neurotoxicity using small-molecule inhibitors that target gingipains may reduce neurodegeneration in Alzheimer's disease [39].

The collagenase activity of *P*. *gingivalis* may also be an important virulence factor since it may participate in gingival tissue breakdown. Indeed, type I collagen makes up approximately 60% of the tissue volume of the periodontium. In this study, we showed that the tart cherry extract inhibits collagen degradation by *P*. *gingivalis*, suggesting that it may contribute to reducing the tissue destructive process. In addition to the action of proteases produced by *P*. *gingivalis*, matrix metalloproteinases secreted by resident and immune cells also participate in this process [40]. We are currently investigating whether the tart cherry extract is also effective in inhibiting the catalytic activity of matrix metalloproteinases.

The adherence of *P*. *gingivalis* to the oral mucosa is the initial step in the invasion of tissues. The ability of *P*. *gingivalis* to adhere to several extracellular matrix proteins, including laminin and type IV collagen, has been previously reported [41]. The present study showed that the tart cherry extract can decrease the adherence of *P*. *gingivalis* to Matrigel^®^, a basement membrane matrix model. Such property may alter the ability of *P*. *gingivalis* to colonize the subgingival sites and consequently reduce their numbers.

The oral epithelial barrier plays a key role in protecting the host against microbial invasion. Tight junction proteins ensure the structural integrity of the barrier by keeping epithelial cells closely attached to one another. They also possess a gate function that modulates the passage of ions and molecules through the paracellular pathway [42]. Groeger *et al*. used specific gingipain inhibitors and gingipain-deficient mutants to show that these *P*. *gingivalis* proteases are involved in the degradation of cell-to-cell junctions and the disruption of the epithelial barrier [43]. Moreover, Andrian *et al*. studied an engineered human oral mucosa model composed of primary epithelial cells and fibroblasts and reported that gingipains are involved in the ability of *P*. *gingivalis* to infiltrate multilayered epithelial cell structures, migrate through the basement membrane, and reach the underlying connective tissue [44]. In a previous study, we found that the integrity of the oral epithelial barrier is strengthened in the presence of tart cherry extracts, as shown by an increase in TEER values and an overexpression of the tight junction proteins ZO-1 and occluding [23]. These two proteins play a key role in establishing cell-to-cell contacts and in maintaining the function of the epithelial barrier and the permeability of the paracellular pathway. Katz *et al*. reported that *P*. *gingivalis* gingipains can degrade epithelial junction transmembrane proteins, including occludin [45]. Given that the tart cherry extract inhibits the proteolytic activity of *P*. *gingivalis*, we hypothesized that it may protect the oral epithelial barrier against bacteria-induced damage. We used an *in vitro* model to show that there is an increase in TEER following a short incubation (≤ 6 h) with *P*. *gingivalis* followed by a decline in TEER. This may reflect a tightening of the host defense barrier until it succumbs to the proteolytic activity of the bacteria. Our results showed that the tart cherry extract can protect the integrity of the oral epithelial barrier despite a challenge with *P*. *gingivalis* by maintaining transepithelial resistance and reducing the flux of FD-4. The protective effect was confirmed by monitoring the expression and distribution of ZO-1 and occludin. This is in agreement with several studies that have reported that quercetin, a major component of the tart cherry extract, has a protective effect on the intestinal epithelial barrier by its ability to induce an increase in TEER values and the overexpression of tight junction proteins [46–48]. Part of the protective effect is also likely related to the ability of the tart cherry fraction to inhibit the Arg- and Lys-gingipains of *P*. *gingivalis*, which can degrade junction proteins.

## Conclusions

Polyphenols have attracted attention in the medical field as potential therapeutic molecules for reducing the use of antibiotics and, as a result, limiting antibiotic resistance among bacteria [49]. The results obtained in the present study suggest that a phenolic extract of tart cherry juice exerts antibacterial activity against *P*. *gingivalis* by reducing its growth and weakening its virulence factors, mostly by inhibiting gingipain activities. Clinical studies are needed to determine whether tart cherry juice consumption or the incorporation of a tart cherry phenolic extract in oral hygiene products (mouthrinses and chewing gums) or slow periodontal-release devices (inserted in diseased periodontal sites) may be potentially used for the treatment of periodontitis.
